# Supplementary material for: How effective is a blended web-based rehabilitation for improving pain, physical activity, and knee function of patients with knee osteoarthritis? Study protocol for a randomized control trial
Source: PLoS One. 2022 May 26;17(5):e0268652. doi: 10.1371/journal.pone.0268652 (PMC9135189; doi:10.1371/journal.pone.0268652)
Supplement: S1 File — (PDF) [file pone.0268652.s002.pdf]

**Τίτλος:** Η επίδραση ενός συνδυαστικού πλαισίου αισθητηριο-κινητικής άσκησης και στρατηγικών διαχείρισης χρόνιου πόνου στον πόνο, τη λειτουργικότητα και τη φυσική δραστηριότητα ασθενών με οστεοαρθρίτιδα γόνατος: Εφαρμογή του στην περιοχή της Δυτικής Αττικής.

**Επισκόπηση Θεωρητικού πεδίου**

Η οστεοαρθρίτιδα γόνατος αποτελεί την πιο κοινή δυσλειτουργία παγκοσμίως και μια από τις πιο συχνές πηγές πόνου στους ηλικιωμένους [1,2]. Αποτελεί μια χρόνια πάθηση με αναντίρρητες κοινωνικές και οικονομικές επιβαρύνσεις. Σε επιδημιολογική μελέτη στις Ηνωμένες Πολιτείες βρέθηκε ότι σε ηλικίες άνω των 45 ετών, η οστεοαρθρίτιδα αφορά το 19,2% των ατόμων, ενώ το ποσοστό σε άτομα ηλικίας άνω των 80 ετών αυξάνει σε 43,7% [3]. Αντίστοιχα στην Ευρώπη, σε μελέτη του Ολλανδικού Ινστιτούτου Δημόσιας Υγείας, το ποσοστό εμφάνισης οστεοαρθρίτιδας γόνατος σε άτομα ηλικίας άνω των 55 αφορά το 15,6% των ανδρών και το 30,5% των γυναικών [4]. Στην Ελλάδα, σύμφωνα με το Ίδρυμα Ρευματολογικών Ερευνών, η επικράτηση της οστεοαρθρίτιδας αφορά το 6% του ενήλικου πληθυσμού [5]. Στην πρόσφατη πανελλήνια επιδημιολογική έρευνα για τις ρευματικές παθήσεις έγινε εκτίμηση των επιπτώσεων της συμπτωματικής οστεοαρθρίτιδας των περιφερικών αρθρώσεων στο κοινωνικό σύνολο και στην εθνική οικονομία της Ελλάδας. Για την εκτίμηση αυτή, και μάλιστα σε σύγκριση με όλες τις άλλες ομάδες παθήσεων στο επίπεδο του γενικού πληθυσμού ενηλίκων, χρησιμοποιήθηκε ο δείκτης της μακροχρόνιας λειτουργικής ανικανότητας. Έτσι, στη μελέτη αυτή βρέθηκε ότι η συμπτωματική οστεοαρθρίτιδα των αρθρώσεων των άνω και κάτω άκρων, προκαλεί σημαντικές δυσμενείς επιπτώσεις στο κοινωνικό σύνολο και στην εθνική οικονομία της Ελλάδος, αφού είναι το τρίτο κατά σειρά συχνότητας αίτιο μακροχρόνιας λειτουργικής ανικανότητας μετά από την ομάδα των υπολοίπων ρευματικών παθήσεων και την ομάδα των καρδιαγγειακών παθήσεων [6]. Η ανάλυση των δεδομένων της έρευνας έδειξε ότι το γυναικείο φύλο, η παχυσαρκία και η ηλικία 50 ετών και πάνω αποτελούν παράγοντες κινδύνου για την οστεοαρθρίτιδα του γόνατος, καθώς και το χαμηλό επίπεδο εκπαίδευσης [6]. Το τελευταίο εύρημα αναφέρεται για πρώτη φορά στη διεθνή βιβλιογραφία. Ο μηχανισμός με τον οποίο το χαμηλό επίπεδο εκπαίδευσης αποτελεί παράγοντα κινδύνου για την οστεοαρθρίτιδα του γόνατος δεν είναι γνωστός. Είναι, πάντως, πιθανό να έχει σχέση με άγνοια των προληπτικών μέτρων για την οστεοαρθρίτιδα του γόνατος μεταξύ των ατόμων με χαμηλό επίπεδο εκπαίδευσης ή/και με επαναλαμβανόμενη επαγγελματική μηχανική επιβάρυνση και καταπόνηση των γονάτων μεταξύ των ατόμων αυτών.

Η άποψη ότι η οστεοαρθρίτιδα αφορά μια φυσιολογική φθορά της άρθρωσης λόγω ηλικίας έχει πλέον αναθεωρηθεί και πλέον κρίνεται ότι η γήρανση του μυοσκελετικού συστήματος αυξάνει απλά την ευπάθεια του ως προς την οστεοαρθρίτιδα, αλλά η ηλικία δεν αποτελεί την αιτία της. Ο πόνος αποτελεί το πρώτο σύμπτωμα της πάθησης. Αρχικά εμφανίζεται μετά από κόπωση και στη συνέχεια μετά από παρατεταμένη ακινησία. Πολλές φορές οι ασθενείς αναφέρουν πως δυσκολεύονται να σηκωθούν από το κρεβάτι ή μετά από παραμονή σε κάθισμα για λίγη ώρα.

Προοδευτικά, ο πόνος γίνεται συνεχής ακόμη και κατά την ανάπαυση και μερικές φορές ξυπνάει τον ασθενή τη νύχτα. Ο πόνος είναι δυνατό να διαρκέσει από μερικές μέρες έως και μήνες. Η αρθρική δυσκαμψία αποτελεί επίσης σύμπτωμα και μπορεί να οδηγήσει ακόμη και σε περιορισμό της κίνησης της άρθρωσης. Λόγω της χρονιότητας της πάθησης αυτής αλλά και των ελλιπών μέτρων διαχείρισης, οι ασθενείς αυτοί βιώνουν πόνο, ο οποίος λαμβάνει χρόνιο χαρακτήρα.

Η πιο διαδεδομένη κατηγοριοποίηση των σταδίων της οστεοαρθρίτιδας ανάλογα με τη σοβαρότητα της, είναι αυτή της Kellgren & Lawrence [7], που σταδιοποιεί τους ασθενείς από 0 ως 4 βαθμούς. Ασθενείς με βαθμό 3 και 4 παραπέμπονται σε χειρουργείο. Σε ποσοστό μόλις 18% παγκοσμίως, οι ασθενείς με οστεοαρθρίτιδα γόνατος έχουν παραπεμφθεί προηγουμένως σε συντηρητικές μεθόδους και φυσικοθεραπεία. Σε ποσοστό 68% οι ασθενείς αυτοί επισκέπτονται Ορθοπαιδικούς χειρουργούς για ενδεχόμενη επέμβαση, οι οποίοι ανάλογα με το στάδιο της εκφύλισης ακτινογραφικά και τον πόνο, τους προωθούν σε χειρουργείο άμεσα ή συνταγογραφούν κάποια είδους φαρμακευτική αγωγή για προσωρινή ανακούφιση και αναμονή για χειρουργείο [8,9]. Συνεπώς, σε ποσοστό 80% οι ασθενείς αυτοί δηλώνουν πως δεν έχουν δεχτεί κάποια σχετική εκπαίδευση ή συμβουλές για τη διαχείριση της οστεοαρθρίτιδας και του χρόνιου πόνου που προκύπτει από αυτή [10]. Σε αυτό το χρονικό διάστημα (που κυμαίνεται από αρκετούς μήνες ή χρόνια) οι ασθενείς βιώνουν καθημερινά πόνο, μείωση στη λειτουργικότητα τους, μειωμένη παραγωγικότητα στην εργασία τους ή και αποχή, καθώς και απουσία από τις κοινωνικές τους υποχρεώσεις. Μελέτες στον Καναδά αλλά και στην Ευρώπη (Ιταλία) κατέγραψαν 2,5% τω εργαζομένων να απέχουν για χρονικά διαστήματα από την εργασία τους λόγω οστεοαρθρίτιδας [11, 12]. Απώτερη συνέπεια αποτελεί η ελλιπής φυσική δραστηριότητα, η σημαντική έκπτωση στην ποιότητα ζωής [13], καθώς και οι σημαντικές οικονομικές επιπτώσεις τόσο κοινωνικά όσο και στο σύστημα υγείας (από τη νοσηλεία και τη συνεχή φαρμακευτική αγωγή) [14]. Ενδιαφέρον αποτελεί το γεγονός ότι οι ασθενείς με οστεοαρθρίτιδα γόνατος που βρίσκονται στη λίστα αναμονής για χειρουργείο παρουσιάζουν χαμηλότερα σκορ σε δείκτες φυσικής υγείας και ποιότητας ζωής από υγιή άτομα ή ακόμη και ασθενείς με καρδιακή ανεπάρκεια [15].

Η επιλεγμένη θεραπεία ασθενών με σοβαρού βαθμού οστεοαρθρίτιδα γόνατος αποτελεί η χειρουργική επέμβαση της αρθροπλαστικής. Μεταξύ 2010/11 καταγράφηκαν 92.000 επεμβάσεις αρθροπλαστικής και 5.800 αναθεωρήσεις (επαναλήψεις) στο Ηνωμένο Βασίλειο. Αντίστοιχα, στην Αμερική, ο αριθμός των ολικών αρθροπλαστικών γόνατος που γίνονται σήμερα, ανέρχεται στο ένα περίπου εκατομμύριο ετησίως ενώ το 2025 θα είναι 2.5 εκατομμύρια και το 2035 αναμένεται στα 3.5 εκατομμύρια. Ο ετήσιος αριθμός επεμβάσεων στην Ελλάδα εκτιμάται σε 20-25.000 αρθροπλαστικές σε γόνατα και ισχίο. Οι επεμβάσεις αυτές έχουν πολλαπλασιαστεί, αφού το 2000 πραγματοποιούνταν περίπου 5-6.000 επεμβάσεις ετησίως. Ο αριθμός αυτός των επεμβάσεων αναμένεται να ανέλθει στις 40.000 ετησίως, λόγω της αύξησης του μέσου όρου ηλικίας και κατ’ επέκταση της ανάγκης των ανθρώπων για μια καλύτερη ποιότητα ζωής, συνεπώς η οικονομική επιβάρυνση

για το Σύστημα Υγείας πολλαπλασιάζεται. Η μέση ηλικία στη χειρουργική επέμβαση αυτή μειώθηκε από 66 σε λιγότερο από 65 έτη σύμφωνα με πρόσφατη μελέτη. Η μείωση στην ηλικία των ασθενών πιθανόν οφείλεται στην αυξανόμενη παχυσαρκία ή στην αυξημένη πρόσβαση στην περίθαλψη, σε μια γενιά που επιθυμεί να διατηρήσει έναν ενεργό τρόπο ζωής στα έτη συνταξιοδότησής τους. Οι ασθενείς που παραπέμπονται τελικά σε χειρουργική επέμβαση αναφέρουν γενικά καλά μετεγχειρητικά αποτελέσματα της αρθροπλαστικής γόνατος. Εντούτοις, το 20% αυτών συνεχίζουν να αισθάνονται πόνο, ο οποίος βρίσκεται σε πλατώ 3-6 μήνες μετά, και άρα χαρακτηρίζεται χρόνιος [16,17]. Σε συστηματική ανασκόπηση και μετα-ανάλυση των Lewis et al, [18] βρέθηκε ότι οι πιο σημαντικοί παράγοντες κινδύνου για την ανάπτυξη χρόνιου πόνου μετεγχειρητικά, είναι η υψηλή ένταση του πόνου προ-εγχειρητικά, καθώς και η ύπαρξη καταστροφικών πεποιθήσεων και συν-νοσηρότητας.

Λαμβάνοντας υπόψη το βιο-ψυχοκοινωνικό μοντέλο του πόνου, οι ασθενείς αυτοί (ακόμη και μέρος αυτών που προχώρησαν σε χειρουργείο) εκτός από τη έκπτωση στην καθημερινή τους λειτουργικότητα, κατέχουν μεγάλο ψυχολογικό φορτίο. Το φορτίο αυτό αναφέρεται συχνά υψηλότερο από άλλους χρόνιους ασθενείς ακόμη και από ασθενείς με σακχαρώδη διαβήτη [19]. Υφίστανται αρνητικές επιπτώσεις στα επίπεδα του άγχους και της ανησυχίας, ενδεχόμενη κατάθλιψη, διαταραχές ύπνου καθώς και κοινωνική απομόνωση [20]. Είναι σημαντικό λοιπό να κατανοηθεί ο μηχανισμός και η πηγή του χρόνιου πόνου στους ασθενείς αυτούς για την αποτελεσματικότερη διαχείριση τους. Ενδιαφέρον προκαλεί το γεγονός ότι η συσχέτιση μεταξύ ακτινογραφικών ευρημάτων και συμπτωμάτων υπάρχει αλλά, δεν είναι ισχυρή. Δεν έχει γίνει ακόμη κατανοητό γιατί πολλές φορές λοιπόν τα ακτινογραφικά ευρήματα της οστεοαρθρίτιδας στο γόνατο δεν συμβαδίζουν με την ένταση και τη χρονιότητα του πόνου [21, 22]. Μια πιθανή εξήγηση αρχικά αποτελεί το γεγονός ότι η οστεοαρθρίτιδα δεν αποτελεί παθολογία μόνο του χόνδρου της άρθρωσης (που απεικονίζεται ακτινογραφικά), αλλά ολόκληρης της άρθρωσης και συνεπώς αλγοϋποδεοχέων (υποδοχέων πόνου) που δεν απεικονίζονται. Δεύτερον, ο πόνος αποτελεί μια εμπειρία πιο πολύπλοκη από καθαρή ιστική / δομική βλάβη που εμπλέκεται με άλλους παράγοντες, όπως ψυχολογικούς που επίσης παίζουν ρόλο στην εμπειρία αυτή. Συνεπώς η φλεγμονή στην άρθρωση και η βλάβη των γύρω ιστών στέλνουν ερεθίσματα πόνου προς τον εγκέφαλο. Ο πόνος όμως όταν υπάρχει για μεγαλύτερα διαστήματα 2-3 μηνών αλλάζει την ψυχολογική και νοητικής κατάσταση των ατόμων που τον βιώνουν [23]. Η κατάθλιψη και η συνυπάρχουσα ανησυχία οξύνουν τον πόνο και επηρεάζουν αρνητικά τη φυσική καθημερινή λειτουργικότητα. Σε κάποιες περιπτώσεις λοιπόν, υπάρχει υπερδραστηριοποίηση των νευρώνων που μεταφέρουν τα ερεθίσματα του πόνου τόσο σε επίπεδο νωτιαίου μυελού όσο και επίπεδου εγκεφάλου με αποτέλεσμα να υπάρχει ευαισθητοποίηση της περιοχής τοπικά στην περιοχή του γόνατος [24]. Αυτοί οι ασθενείς αναφέρουν πόνο περισσότερο διάχυτο από την άρθρωση του γόνατος. Παράλληλα όμως, συνυπάρχει κεντρική ευαισθητοποίηση και λόγω της πλαστικότητας στο νευρικό σύστημα, ο εγκέφαλος συνεχίζει να στέλνει μηνύματα πόνου ακόμη και αφού το αρχικό ερέθισμα του πόνου έχει επιλυθεί [25,26]. Πλέον υπάρχουν ερευνητικά δεδομένα που επιβεβαιώνουν τόσο ψυχολογικά όσο και νευρο-απεικονιστικά στους

ασθενείς αυτούς πόνο με κεντρική ευαισθητοποίηση [27]. Συνεπώς σύγχρονες μελέτες πρόσφατα έχουν στραφεί σε θεραπευτικές προσεγγίσεις που αξιολογούν και στοχεύουν στην διαχείριση του χρόνιου πόνου, τη βελτίωση της λειτουργικότητας, της φυσικής δραστηριότητας αλλά και την τροποποίηση των ψυχολογικών και γνωσιακών παραμέτρων όπως πεποιθήσεων, προσδοκιών σε ασθενείς με οστεοαρθρίτιδα γόνατος.

Η άσκηση και γενικά η συμβουλή για ενίσχυση της φυσικής δραστηριότητας προσφέρει οφέλη στη μείωση της έντασης του χρόνιου πόνου, στην βελτίωση της λειτουργικότητας και της πνευματικής υγείας σύμφωνα με πρόσφατη συστηματική ανασκόπηση της Cochrane [28]. Συγκεκριμένα, η θεραπευτική άσκηση της αισθητηριο-κινητικής εκπαίδευσης αποτελεί μια εξειδικευμένη προσέγγιση που στοχεύει στα ελλείμματα των ασθενών, όπως η μειωμένη λειτουργικότητα, η μειωμένη μυϊκή δύναμη, ισορροπία και ιδιοδεκτικότητα, καθώς και ο αυξημένος ο κίνδυνος πτώσεων [29]. Αυτή η μορφή άσκησης έχει φανεί να βοηθά σημαντικά τους ασθενείς στα ελλείμματα αυτά, αλλά και στον πόνο στην οστεοαρθρίτιδα γόνατος [30,31]. Σύμφωνα με την πρόσφατη αρθρογραφία [32, 33] αλλά και τα ευρήματα της διδακτορικής διατριβής που αποτελεί βάση αυτής της πρότασης [34-36], άσκηση με ερεθίσματα αισθητηριο-κινητικής φύσης (δηλαδή ερεθίσματα που στοχεύουν στην διατάραξη ισορροπίας και προαγωγής ευκινησίας και νευρομυϊκού συντονισμού) επιδρά θετικά στον πόνο, τη λειτουργικότητα, την ισορροπία, τη μυϊκή δύναμη αλλά και σε αυτό-αναφερόμενα εργαλεία σωματικής και πνευματικής υγείας μετά την αρθροπλαστική σε ασθενείς με οστεοαρθρίτιδα γόνατος. Η άσκηση σύμφωνα με τα ευρήματα αυτά μπορεί να εκτελείται ακόμη και σε περιβάλλον σπιτιού χωρίς εξειδικευμένο εξοπλισμό. Σε μελέτη απόψεων Ελλήνων φυσικοθεραπευτών σχετικά με τις στρατηγικές αποκατάστασης ασθενών με οστεοαρθρίτιδα γόνατος μετά την χειρουργική παρέμβαση, φάνηκε ότι υπάρχει σχετική σύμπνοια στο πρόγραμμα άσκησης που ακολουθείται, το οποίο όμως στοχεύει κυρίως στην αποκατάσταση του εύρους τροχιάς της άρθρωσης και της μυϊκής δύναμης αντίστοιχα [37]. Επιπλέον, δήλωσαν πως δεν παραπέμπονται σε αυτούς ασθενείς προεγχειρητικά για προετοιμασία, αλλά και μετεγχειρητικά μεγάλο ποσοστό ασθενών μετά το εξιτήριο από το νοσοκομείο πηγαίνουν σπίτι χωρίς συμβουλές για άσκηση από τους Ορθοπαιδικούς χειρουργούς [37]. Αντίστοιχες μελέτες διεθνώς φανερώνουν παρόμοια ευρήματα [38], με αποτέλεσμα τα ελλείμματα των ασθενών αυτών σχετικά με την λειτουργικότητα τους , την αυτοπεποίθηση που απορρέει από την ισορροπία και την κινητικότητα τους αλλά και η εμπειρία του πόνου να μην αντιμετωπίζονται στοχευμένα. Συνεπώς, μια περισσότερο ολιστική προσέγγιση που παράλληλα με την θεραπευτική άσκηση αυτή να στοχεύει στην ενίσχυση της εν γένει φυσικής δραστηριότητας, στην εκπαίδευση των ασθενών για τη διαχείριση του πόνου, αλλά και στη συμπεριφορά των ασθενών σε σχέση με αυτόν, τις πεποιθήσεις και τα συναισθήματα τους, κρίνεται περισσότερο ολοκληρωμένη και αναγκαία [39].

Η γνωσιακή συμπεριφορική θεραπεία αποτελεί μια προσέγγιση που στοχεύει στην τροποποίηση της συμπεριφοράς ατόμων με τη χρήση συμβουλευτικής προκειμένου να διαχειριστούν ενεργά προσωπικά, εσωτερικά ζητήματα καθώς και ζητήματα

σκέψης, συναισθήματος, συμπεριφοράς και αλληλεπίδρασης. Απώτερος στόχος αποτελεί η βελτίωση της συνολικής ποιότητας της ζωής, μέσα από την ανάπτυξη από τους ίδιους δεξιοτήτων, προκειμένου να αυτο-διαχειρίζονται μη παραγωγικές πεποιθήσεις, προσδοκίες και σκέψεις γύρω από προκλήσεις της ζωής τους, όπως άγχος, άνοια, πένθος, πόνος. Αποτελεί πλέον μια από τις συνήθεις ενεργητικές μεθόδους αποτελεσματικής διαχείρισης ασθενών με χρόνιο πόνο [40,41]. Σύμφωνα με αυτή, εφαρμόζεται μια βραχύχρονη εκπαίδευση των ασθενών σε σχέση με τη διαχείριση του πόνου αποτελεί μια προσέγγιση που στηρίζεται σε 2 σκέλη: α) ενημέρωση του ασθενή σε σχέση με το πως ψυχοκοινωνικοί (συναισθήματα φόβου, αυξημένη ανησυχία, αδυναμία συγκέντρωσης, μειωμένη κοινωνική υποστήριξη) και περιβαλλοντικοί παράγοντες επηρεάζουν την πόνο που βιώνει και την λειτουργικότητα του στην καθημερινότητα, και β) συστηματική εκπαίδευση στρατηγικών (σταδιακή δραστηριοποίηση ασθενή, επίλυση προβλημάτων, εκπαίδευση χαλάρωσης) προκειμένου να διαχειρίζεται αποτελεσματικά τα συμπτώματα του. Επιπρόσθετα εφαρμόζονται μέθοδοι ενημέρωσης του ασθενή για την βιολογία, νευροφυσιολογία και επεξεργασία του πόνου, και επομένως πως η αντίληψη του αυτή και συμπεριφορά σε σχέση με τον πόνο επηρεάζει τις εμπειρίες του, τη συμπεριφορά του και μετέπειτα και την ποιότητα ζωής του. Συγκεκριμένα για ασθενείς με χρόνιο πόνο, νευρο-μυοσκελετικής αιτιολογίας (όπως πχ. Οσφυαλγία, οστεοαρθρίτιδα) εφαρμόζονται στα πλαίσια της φυσικοθεραπείας, στοχευμένες προσεγγίσεις που απευθύνονται τόσο σε επαγγελματίες υγείας όσο και απευθείας σε ασθενείς προκειμένου να διδαχτούν στρατηγικές αντιμετώπισης του χρόνιου πόνου. Μάλιστα πρόσφατα προκειμένου να γίνουν ευρέως διαδομένες οι προσεγγίσεις αυτές στους επαγγελματίες υγείας και τους ασθενείς, παρέχονται οργανωμένα πλαίσια με σεμιναριακού τύπου υλικό που περιλαμβάνουν συμβουλές για άσκηση και στρατηγικές αυτοδιαχείρισης πόνου έχουν επιχειρηθεί με πρωτοβουλίες Πανεπιστήμιων. Συγκεκριμένα με πρωτοβουλία του Πανεπιστημίου του Oxford στο Ηνωμένο Βασίλειο δημιουργήθηκε το πρόγραμμα Back Skills Training (BeST Programme) προκειμένου να ενημερώσει και αντιμετωπίσει προβλήματα πόνου σε ασθενείς με οσφυαλγία [42]. Το 2013 επίσης έπειτα από μελέτη που διενεργήθηκε από το Πανεπιστήμιο της Νότιας Δανίας καθιερώθηκε το πρόγραμμα GLA:D (Good Life with osteoArthritis in Denmark programme) [43] με επιτυχημένα αποτελέσματα για ασθενείς με οστεοαρθρίτιδα. Το πρόγραμμα αυτό διαδόθηκε σταδιακά στην Αυστραλία και τον Καναδά προσφέροντας εύκολα προσβάσιμο ηλεκτρονικό υλικό (μέσω ηλεκτρονικής πλατφόρμας και εφαρμογών κινητού τηλεφώνου (πχ. PAIN trainer) [44] και παράλληλα ελάχιστη επίβλεψη από φυσικοθεραπευτή, στοχεύοντας στην εκπαίδευση των ασθενών σχετικά με την εμπειρία του πόνου και στρατηγικές αντιμετώπισής του, που εμπεριέχουν άσκηση και πρόληψη.

Συνεπώς, η ενσωμάτωση προσεγγίσεων για την διαχείριση του πόνου (όπως η γνωσιακή συμπεριφορική θεραπεία ή η εκπαίδευση της νευρο-επιστήμης του πόνου) στα πλαίσια της φυσικοθεραπευτικής άσκησης αποτελεί μια περισσότερο ολιστική προσέγγιση που τελευταία υπάρχουν ερευνητικές ενδείξεις για τη αποτελεσματική διαχείριση ασθενών με οστεοαρθριτιδα γόνατος. Φαίνεται να είναι αποτελεσματική τόσο για την αντιμετώπιση του πόνου όσο και για την ενίσχυση της φυσικής δραστηριότητας [45-47], όπως προτείνεται από τον Παγκόσμιο Οργανισμό Υγείας.

Για τους ασθενείς με χρόνιο οστεοαρθριτικό πόνο έχει τεκμηριωθεί ερευνητικά η προσπάθεια εξισορρόπησης από πλευράς των θεραπευτών της χρήσης τεχνικών δια χειρός και μη (προσεγγίσεις όπως οι προαναφερθείσες) στην κλινική πράξη για την αποτελεσματικότερη διαχείριση τους [48]. Μάλιστα διεξάγονται πιλοτικές μελέτες προκειμένου οι προσεγγίσεις αυτές να εφαρμοστούν ακόμη και σε ασθενείς με μειωμένη πρόσβαση σε δομές υγείας από απόσταση [49,50].

**Ερευνητικοί Στόχοι:**

Κρίνεται αναγκαίο τόσο οι παραπάνω ερευνητικές ενδείξεις όσο και αυτές που προέκυψαν από την διδακτορική διατριβή από την οποία εφορμά αυτή η πρόταση, να εφαρμοστούν σε ένα ανάλογο θεραπευτικού πλαισίο στην Ελληνική κοινωνία. Στα πλαίσια λοιπόν της άμεσης εφαρμογής τεκμηριωμένης γνώσης και προσφοράς του έργου του Πανεπιστημίου Δυτικής Αττικής στην κοινωνία, στόχος αποτελεί σε πρώτη φάση το φυσικοθεραπευτικό αυτό πλαίσιο να απευθυνθεί στους κατοίκους των Δήμων της Δυτικής Αττικής. Η επιλογή της Δυτικής Αττικής, «γνωστή και ως πίσω αυλή της πρωτεύουσας» έγινε λόγω του ότι αποτελεί ένα πολεοδομικό ιστό σχετικά υποβαθμισμένο σε σχέση με τις υπόλοιπες συνοικίες της Αττικής από πλευράς υποδομών. Συνεπώς, στόχος είναι μέσα από αυτήν την εφαρμογή αυτή η συνεργασία του Πανεπιστημίου με την Πολιτεία προκειμένου να εφαρμοστεί ένα πλαίσιο αμοιβαίας αλληλεπίδρασης και προσπάθειας αναβάθμισης της φροντίδας και ποιότητας ζωής για τους κατοίκους αυτών των Δήμων.

Σκοπός αυτής της μετα-διδακτορικής μελέτης θα είναι η διερεύνηση της αποτελεσματικότητας ενός συνδυαστικού πλαισίου αισθητηριο-κινητικής άσκησης και εκπαίδευσης στρατηγικών διαχείρισης του πόνου στη λειτουργικότητα, στα επίπεδα φυσικής δραστηριότητας, ποιότητας ζωής, στη διαχείριση πόνου και άγχους σε ασθενείς με χρόνιο οστεοαρθριτικό πόνο στο γόνατο. Το πλαίσιο αυτό θα ενθαρρύνει μέσα από οργανωμένη και συστηματική άσκηση τη φυσική δραστηριότητα σε κατάλληλους χώρους στην Δυτική Αττική (πχ. διαδρομή σε Πάρκο αναψυχής). Παράλληλα θα περιλαμβάνει εκπαίδευση και άσκηση στο περιβάλλον του σπιτιού τους με μικρή φυσική επίβλεψη και ηλεκτρονική υποστήριξη προκειμένου να ενθαρρύνει την αυτοδιαχείριση και τη σωματική και πνευματική υγεία, αλλά και αγγίξει και ασθενείς με μειωμένη πρόσβαση.

**Ερευνητική Υπόθεση:**

Μηδενική Υπόθεση: Δεν υπάρχει διαφορά στα επίπεδα λειτουργικότητας, ποιότητας ζωής και πόνου μετά από την εφαρμογή ενός συνδυαστικού φυσικοθεραπευτικού πλαισίου αισθητηριο-κινητικής άσκησης και συμβουλευτικής για τον πόνο μεταξύ της πειραματικής ομάδας και ομάδας ελέγχου ασθενών με χρόνια οστεοαρθρίτιδα.

Εναλλακτική Υπόθεση: Υπάρχει διαφορά στα επίπεδα λειτουργικότητας, ποιότητας ζωής και πόνου μετά από την εφαρμογή ενός συνδυαστικού φυσικοθεραπευτικού

πλαισίου αισθητηριο-κινητικής άσκησης και συμβουλευτικής για τον πόνο μεταξύ της πειραματικής ομάδας και ομάδας ελέγχου ασθενών με χρόνια οστεοαρθρίτιδα.

**Μεθοδολογία:**

Στη μελέτη αυτή θα συμμετέχουν ασθενείς με χρόνιο οστεοαρθριτικό πόνο (σύμφωνα με τις οδηγίες OARSI Guidelines for Osteoarthritis Care and Management in Adults), ηλικίας 45 ετών και άνω, από την περιοχή της Δυτικής Αττικής, οι οποίοι να ενδιαφέρονται να ενταχθούν στο πλαίσιο αυτό. Η ακτινογραφική διάγνωση δεν θα είναι αναγκαία για την διάγνωση της οστεοαρθρίτιδας γόνατος. Οι συμμετέχοντες θα επιστρατευθούν με πρωτοβουλία και πρόσκληση του Πανεπιστημίου Δυτικής Αττικής και συνεργασία των γειτονικών Δήμων από κοινωνικές δομές και δομές υγείας. Το σχέδιο μελέτης θα αφορά ασθενείς με χρόνιο οστεοαρθριτικό πόνο στο γόνατο οι οποίοι θα συγκριθούν με ομάδα ελέγχου που θα απαρτίζεται από υγιή άτομα αντίστοιχης ηλικίας σε βάθος χρόνου. Κριτήρια αποκλεισμού θα αφορούν ασθενείς με άλλα προβλήματα δυσλειτουργίας γόνατος όπως ρευματοειδή αρθρίτιδα, κατάγματα, όγκοι, ινομυαλγία.

Το θεραπευτικό πλαίσιο της πειραματικής ομάδας θα περιλαμβάνει 6-8 συνεδρίες στρατηγικών εκπαίδευσης πόνου και πεποιθήσεων και συμπεριφοράς γύρω από αυτόν. Παράλληλα και σύμφωνα με τις πρόσφατες οδηγίες για φυσική δραστηριότητα, οι ασθενείς θα ασκούνται για 150 λεπτά την εβδομάδα σε μέτριας έντασης άσκηση που θα περιλαμβάνει αερόβια άσκηση, λειτουργικές ασκήσεις και ασκήσεις αισθηριο-κινητικής εκπαίδευσης (2008 Physical Activity Guidelines for Americans).

Οι συμμετέχοντες θα αξιολογούνται και η επιδόσεις τους θα καταγράφονται στην έναρξη του πλαισίου 3 και 6 μήνες μετά. Στη διάρκεια του πλαισίου θα συμπληρώνεται από τους συμμετέχοντες ημερολόγιο φυσικής δραστηριότητας και συμμετοχής στο πλαίσιο προκειμένου να αξιολογείται η συμμετοχή και συμμόρφωση τους στο πρόγραμμα. Προτεινόμενα μέτρα έκβασης θα περιλαμβάνουν λειτουργικές δοκιμασίες (6-min walk test), αυτό-αναφερόμενες κλίμακες λειτουργικότητας και φυσικής δραστηριότητας (Knee Injury and Osteoarthritis Outcome Scale), κλίμακες πόνου (Numeric Pain Rating Scale) και κλίμακες γενικής υγείας και άγχους/κατάθλιψης (SF-12, Patient health questionnaire 9, Coping Strategy Indicators).

- Το 6/λεπτο τεστ βαδίσματος (six-minute walk test).

Η εξάλεπτη δοκιμασία περπατήματος περιγράφει, με έμμεσο τρόπο, το επίπεδο της αερόβιας αντοχής και έχει ως σκοπό να καλύψει ο εξεταζόμενος να καλύψει όσο το δυνατόν γρηγορότερα μεγαλύτερη απόσταση μέσα σε 6 λεπτά. Η διαδρομή που θα διανυθεί είναι σημαδεμένη με πόλους και σχεδιασμένη στο έδαφος.

- Knee injury and Osteoarthritis Outcome Score (KOOS)

Το ερωτηματολόγιο KOOS είναι ένα ερωτηματολόγιο αξιολόγησης λειτουργικότητας γόνατος. Αξιολογεί πόνο λειτουργικότητα σε καθημερινές δραστηριότητες, ποιότητα ζωης, ικανότητα συμμετοχής σε σπορ. Χρησιμοποιείται σε ασθενείς μετά από τραυματισμό γόνατος, σε οστεοαρθρίτιδα γόνατος ή έπειτα από χειρουργείο. Στην παρούσα διασκευή χρησιμοποιήθηκαν ασθενείς μετά από αρθροπλαστική γόνατος.

- Αριθμητική κλίμακα (Numerical Rating Scale-NRS)

Αποτελείται από μία απλή κλίμακα από το μηδέν έως το πέντε ή το δέκα, όπου το μηδέν αντιστοιχεί σε ανύπαρκτο πόνο και το πέντε ή το δέκα αντιστοιχεί στο χειρότερο δυνατό πόνο. Ο ασθενής επιλέγει τον αριθμό που ανταποκρίνεται καλύτερα στην ένταση του πόνου του. Η συγκεκριμένη κλίμακα είναι επίσης απλή και εύχρηστη, καθώς μπορεί να χρησιμοποιηθεί από ασθενείς διαφόρων ηλικιών και μορφωτικών επιπέδων όπως και από αυτούς που δυσκολεύονται να συνεργαστούν λόγω έντονου πόνου αφού μπορεί να χρησιμοποιηθεί και προφορικά. Πρόκειται για μία κλίμακα αξιόπιστη με πολύ καλή ευαισθησία στην ανίχνευση μεταβολών στην ένταση του πόνου.

- Patient Health Questionnaire-9

  Η PHQ-9 είναι κλίμακα κατάθλιψης εννέα στοιχείων που αφορά τη διάγνωση της κατάθλιψης, καθώς και την επιλογή και την παρακολούθηση της θεραπείας.

- SF-12 (Short Form 12 Health Survey Questionnaire )

Το ερωτηματολόγιο της ποιότητας ζωής, SF-12 αποτελείται από 12 ερωτήσεις που αξιολογούν διαστάσεις αφορούν την σωματική υγεία και πενυματική/ψυχική υγεία.

Ηθική έγκριση της μελέτης θα αναζητηθεί από την Επιτροπή Ηθικής και Δεοντολογίας του Πανεπιστημίου Δυτικής Αττικής.

**Χρονοδιάγραμμα Εργασιών:**

| | Gant Chart | | | | |
|---|---|---|---|---|---|
| **Μήνες** | **0-3** | **6** | **12** | **18** | **24** |
| **Σχεδιασμός Ερευνητικού Πρωτοκόλλου Ανασκόπηση Αρθρογραφίας & Σύσταση Υλικού** | | | | | |
| **Έγκριση Επιτροπής Ηθικής & Δεοντολογίας** | | | | | |
| **Συλλογή Δεδομένων** | | | | | |
| **Ανάλυση Δεδομένων** | | | | | |
| **Συγγραφή Διατριβής** | | | | | |

**Αναμενόμενα αποτελέσματα:**

Τα ευρήματα της μελέτης θα ενημερώσουν την φυσικοθεραπευτική κλινική πρακτική ως προς το κατά πόσο ένα συνδυαστικό πλαίσιο διαχείρισης πόνου και άσκησης με φυσική και ηλεκτρονική υποστήριξη είναι εφικτό και αποτελεσματικό να εφαρμοστεί σε ασθενείς με οστεοαρθριτικό πόνο προκειμένου να βελτιώσει τα επίπεδα πόνου, φυσικής δραστηριότητας και ποιότητας ζωής.

Translation of Study Protocol submitted to IKY (State Foundation Scholarship, Greece)

Applicant code: 18079

# Title: How effective is a blended web-based rehabilitation for improving pain, physical activity, and knee function of patients with knee osteoarthritis? Study protocol for a randomized control trial"

## Introduction

Osteoarthritis of the knee (KOA) is a prevalent and debilitating condition characterised by pain, and difficulty performing normal daily activities (ADL) such as walking, which can lead to psychological distress, reduced health-related quality of life (QoL) and absenteeism from work worldwide [1]. Primary care clinicians (i.e. family doctors, general practitioners), where KOA is mostly diagnosed and managed, lack knowledge on conservative options and access to services so as to guide treatment [2]. Therefore, in several countries, current care for KOA patients is often not consistent with the clinical guidelines [3,4] recommending strengthening exercises, increase in low impact physical activity and weight management if needed, for this clinical population [5,6,7,8,9]. Alternatively drug therapy and surgical interventions are over-emphasized and add an economic burden to the Health Care System [5, 8, 10].

Chronic conditions such as KOA significantly have an unfavorable impact on patients' physical activity (PA), behavior towards movement and consequently QoL [11, 12]. Due to the chronic nature of the KOA, education and awareness of the condition (i.e. expectations, advice on how to best manage potential flare-ups, benefits of exercise and activity, adjustment of lifestyle) is important. Interventions that foster appropriate lifestyle behavioral change, particularly around PA, are also important for such chronic diseases. Current PA guidelines suggest that people with OA should be as physically active as their abilities and condition allows [13]. Experiencing benefits from exercise and physical activity participation, has shown to help shaping positive beliefs and motivated individuals towards maintaining activity [14]. Therefore, this clinical population should be aware of how to self-manage their condition, in order to avoid restriction on their ADL, as this can lead to a vicious circle of inactivity, muscle weakness, weight gain, limited socializing, depression, pain increase and functional impairment [15, 16]. Another important issue, is that this clinical population often lacks access to community services (due to rural or weakly structured areas, financial resources or age-related transfer difficulties) that include different types of exercise, advice. Nowadays, with the rapid changes and measures COVID-19 has enforced, the need for home-based self-managed care or outdoor open air training options are essential for health and safety precaution. It is critical to identify and address all the aforementioned concerns early on, and develop effective strategies that facilitate uptake of recommended KOA management practice.

In accordance with a biopsychosocial approach to the management of chronic pain [17] both physical and psychological impairments should be addressed in people with KOA. Exercise therapy is effective in reducing patients' levels of pain and increasing their physical activity [18]. Unfortunately, the face-to-face contacts with a physical therapist are costly and a burden for the health care budget. Moreover, despite the positive effects on symptoms, exercise-focused programmes do not promote sustained behavior change. Combining exercise and self-management (education and advice strategies) might enhance their separate benefits. However, dynamic facilitators in engaging and sustaining exercise and PA are support by healthcare professionals and community, training and awareness on the condition, encompassing behavioral interventions, purposeful, enjoyful and flexible modes of PA [14]. Web-based rehabilitation could have the potential to increase access in structurally weak areas, where appropriate healthcare infrastructure is lacking and offers are missing and also be a more cost-effective option. Moreover, a great advantage of this type of rehabilitation is that it can be performed at a self-determined time, according to its individuals' schedule, and in a home-based environment, which could enhance adherence to the programme and resolve transportation and cost issues. There is mounting evidence that orthopedic technology-assisted rehabilitation has a positive impact on various clinical conditions [19-22]. Previous studies have noted that a tele-physiotherapy program for KOA offers comparable benefits in physical function and pain [23].

Given the above, there is a need for a program that encompasses physical and behavioral elements, with purposeful and flexible PA, as well as interplay of self-management and healthcare/social support strategies to promote a good fit for the care of KOA. Thus, all exercise based programs in order to be adopted in the daily routine need to encompass rea-life outdoor activity. Walking as a flexible, purposeful exercise is related to living an active and independent life and is therefore important for achieving pain relief and normal daily functioning [24]. Walking difficulties seem to influence patients' future health. Unreleaved pain should be assessed by clinicians as another factor that may lead to patients' decrease of walking ability over time [25]. Walking difficulties were associated with higher mortality rates 10 years after knee replacement [26]. In addition, walking difficulty and shorter walking distance predicted higher health care costs and disability, as well as poorer self-rated health in the general population [27-28].

## Objectives

This project primarily aims to compare the efficacy of a 6-week web-based rehabilitation program of exercise, advice and enhanced outdoor structured PA to manage pain and physical function in individuals with KOA compared to outdoor PA program alone. The secondary aims of the study are to compare the efficacy of these programs on functional performance, psychological parameters, QoL, PA levels to examine whether outcomes are maintained at mid-term over a 3-month follow up period.

## Primary hypothesis

H1: A 6-week web-based rehabilitation program of exercise, advice enhanced with structured outdoor activity will be more efficacious in improving pain and self-reported

physical function than a 6-week enhanced outdoor activity program alone immediately following the intervention.

H2: A 6-week web-based rehabilitation program of exercise, advice enhanced with structured outdoor activity will be more efficacious in improving pain and self-reported physical function than a 6-week enhanced outdoor activity program alone at 12 weeks.

H3: A 6-week web-based rehabilitation program of exercise, advice enhanced with structured outdoor activity will be more efficacious in improving psychological function, functional performance, QoL and physical activity levels than a 6-week enhanced outdoor activity program alone at 12 weeks.

## Trial design

This will be an exploratory, parallel group, 2-arm prospective randomised controlled trial.

## Methods

### Study Setting

A sample of 60 patients will be recruited from the community of municipalities of West Attica in Athens, Greece. The selection of west Attica municipalities was based on the weak, relevantly neglected infrastructure this region holds as well as the poorer financially resources invested. Therefore, physiotherapy local community services will be of outmost importance in clinical populations with chronic conditions that encounter these difficulties. An open invitation will be published from the administration office of the municipalities and participants will register by providing their contact details. Participants will be given specific information by physiotherapists working in the municipalities, not involved in the trial, detailing the intervention they would receive. Persons interested in participating will sign up and will provide their contact details. The investigator (physiotherapist-AK) will telephone the participants interested and provide a verbal explanation of the trial. All participants will provide written informed consent to the investigator who will perform all assessments sessions; eligibility criteria will be checked and a baseline assessment will be arranged for those eligible and willing. At this assessment, written consent will be obtained and a medical history will be taken. Patient characteristics i.e. age, sex, height, weight, educational level, location of OA, disease duration and the presence of comorbidities will be assessed at baseline.

The trial has been prospectively registered in the ISRCTN clinical trial registry (ISRCTN12950684) on the 27th of September 2020. Ethics approval was granted the Research Ethics Committee of University of West Attica, Greece (49238/09-07-2020). This research study is co-financed by Greece and the European Union (European Social Fund-ESF) through the Operational Programme «Human Resources Development, Education and Lifelong Learning» in the context of the project “Reinforcement of Postdoctoral Researchers - 2nd Cycle” (MIS-5033021), implemented by the State Scholarships Foundation (IKY).

### Eligibility Criteria

To be eligible, participants must fulfill the following criteria:

i. Aged ≥ 45 years; ii. Clinical diagnosis of Knee OA (Kellgren and Lawrence ≥ Grade 1-3); iii. Knee pain for ≥ 3 months; iv. Reporting average knee pain in the last week ≥ 3 on an 11-point Numeric Pain Rating Scale (NPRS); v. ability to write and speak Greek; xi. able to use/access computer or tablet and have access to the internet.

The exclusion criteria are:

Knee surgery including arthroscopy within the past 6 months; ii. Awaiting or planning any back or lower limb surgery within the next 12 months; iii. Current or past (within 3 months) oral or intra-articular corticosteroid use; iv. Systemic arthritic conditions such as rheumatoid arthritis or gout; v. Physiotherapy, chiropractic or acupuncture treatment or exercises specifically for the knee within the past 6 months; vi. Inability to walk unaided as this is necessary for some of the physical testing; vii. Medical condition precluding safe exercise such as uncontrolled hypertension or heart condition; viii. Self-reported psychiatric history such as schizophrenia or cognitive impairment precluding safe compliance to the program; ix. Neurological condition such as Parkinson's disease, multiple sclerosis or stroke.

Interventions

During the first face-to-face session (week 1), the physiotherapist (researcher- MM) will provide information about OA, the importance of PA and the relation of PA with pain. This introductory session will be scheduled for all participants (both groups) to get familiarized with the webpage environment. Moreover, the journey route of the outdoor walking program appropriate for each participant will be selected and mutually agreed. To ensure consistency in content and delivery, the same physiotherapist (who had 16 years of postgraduate clinical experience) will devise, supervise, and progress all sessions of all participants. Participants will continue taking their usual medications and other non-surgical treatments to manage their KOA, and use normal assistive devices such as a cane if needed. Weekly telephone supervision to encourage adherence and resolve any issues i.e. potential overload or motivation. In week 7, a second face-to-face session will take place. To assess patients' progress and encouragement to continue for another 6 weeks will be promoted by the researcher. An online report containing a summary of website-visits and patients' experiences with the exercises and outdoor activity will be sent to the researcher. A final face-to-face appointment will take place in week 12 by the researcher to support and encourage patients to maintain a physically active lifestyle. A final assessment will be performed will be performed by the investigator.

Web-based and outdoor physical activity program (WB-OPA)

Participants randomized to web-based rehabilitation (based on ESCAPE-knee pain resources, after having taken relevant approval) participated in an exercise-based rehabilitation program designed to improve function by integrating exercise, education, and self-management strategies to dispel inappropriate health beliefs, alter behavior, and encourage regular PA. In the introductory session individual passwords will be provided to participants in this group in order to register and have access to the rehabilitation regime. Participants will be instructed to attend 12 sessions, for 6 weeks, twice weekly. More specifically, each

week, participants will be encouraged to attend a 5-10 min video advisory session by the physiotherapist, on a specific topic, advising and suggesting simple coping strategies. The advisory sessions include information on OA related topics, risk factors, clinical manifestations, prognosis, feasible non-pharmacological therapies, like ice, dietary control, pacing, goal setting, and overcoming psychological distress. Then, for 35–40 minutes each participant will perform a simple exercise regimen based on pre-recorded video exercises to address their disabilities and progressed this as they improve. The exercises aim to train elements of joint flexibility, muscle strength, balance, and muscle endurance. Table 1 presents the outline of the intervention procedure.

In addition to the web-based exercise program, participants in the intervention group will be prescribed to perform thrice weekly a pre-determined walk of 500-800m (a walking dose of 70 min per week), according to their individual potential. The journey routes will be selected based on a distance of 500-850m, and contained safe sidewalks, comfortable, smooth and enjoyable paths (local green parks, pedestrian shopping malls, sports courts etc.). This journey route will be selected and agreed in the introductory face-to face session and a relevant map will be provided. Walking programs are known to have beneficial effects on knee pain and function for people with mild to moderate knee OA [29]. The weekly dose will be completed for 6 weeks in the community. The participant will be instructed to walk at a moderate level of intensity determined by the Rate of Perceived Exertion Scale (0–10) where level 3 = "I am still comfortable but am breathing a little harder". No formal instructions on warming up or stretching were provided. The weekly dose of 70 min will be completed in the community; in separate sessions provided each session was at least 10 min duration.

Usual care

Participants randomized to usual care (the pragmatic control arm) will be guided to engage PA in their daily routine. A walking-based exercise program aiming to improve pain and functional limitation will be prescribed. In the first session, they will be introduced to the same webpage, where the general information on KOA will be available for education purposes and will be guided to select a journey route from a number of journey routes developed within the community. They will be encouraged to walk this journey 5 times weekly and again a relevant map of the route will be provided with analytic description, photos of key corners, benches for rest etc.

Strategies to improve adherence to interventions

To increase the likelihood of adherence to the intervention, the following behavioural change techniques and strategies will be used. First, each participant will have a planning session with a physiotherapist of up to 30 min to plan the location, day and time of day for each walk, and reinforce that each walk was moderate intensity in at least a 10 min. Second, regular physiotherapy supervision and monitoring each week with regular phone calls based on patient preference will be offered. If any complication or query should arise, participants could discuss it in the weekly phone calls or contact directly the physiotherapist, and they will be informed of the procedure to follow. Third, each participant will wear a pedometer and record the number of steps taken and time spent walking during each session in a

logbook. Fourth, participants will be encouraged to engage social supports such as walking with a friend, family member if they chose to.

Participants in both groups will be free to withdraw from the study at any time, but the withdrawal rates and reasons will be recorded.

A simply-worn pedometer will be given to all participants in order to quantify walking and record their steps/day) in a diary. While standing, a pedometer will be clipped to the participant's waistband on either the left or right hip.

Telephone-based supervision will be offered in weekly basis in order to monitor safe progress for both groups.

All participants will complete a logbook to record the number of minutes spent walking and number of steps using a pedometer for each walking session over the 12 weeks (Table 2). They will also describe any changes to their usual care of their knee OA and any problems with their knee while doing their walking program each week. The physiotherapist will monitor the participant's logbook at each weekly supervisory session. For the intervention group, the diary will include logs where they will be instructed to record apart from the number of steps/day the frequency and time spent when they will perform their web-based exercise program.

Provisions for post-trial care

After completion of the 6-week program, participants will be discharged with encouragement to perform the home-based exercise regime and continue improving PA levels, especially with the walking journey routes, but will not receive any additional intervention as part of the program for the following 6 weeks till the final follow-up.

Outcomes

Primary outcome measures

Outcome measures have been selected based on those recommended for clinical trials of OA [30-31]. The primary outcomes are change in self-reported pain and physical function at 6 and 12 weeks. The primary and secondary outcomes will be collected at baseline and post-intervention (week 6) and final follow-up (week 12) by an assessor blinded to group allocation.

a) Physical function: this will be self-assessed using the Knee Injury Osteoarthritis Outcome Score (KOOS) Likert version.

b) Pain: Average knee pain over the previous week measured by a valid and reliable Numerical Pain Rating Scale pain with terminal descriptors 0 (no pain) and 10.

Secondary outcome measures

c) Physical Function:

- The 30-second Chair Stand test (30 CTS) provides a direct, objective measure of physical function.
- The timed up and go (TUG) test evaluates walking speed and mobility [36]. has been found as valid and reliable in KOA patients [37].

d) Physical activity level:

Habitual PA will be measured in three ways, one using a questionnaire, the second using a scale and a third using a pedometer.

- The Modified Baecke Physical Activity Questionnaire (mBQ) is one of the most widely used questionnaires to assess physical activity in both healthy individuals and patients. The Greek version of the mBQ [38]
- The UCLA scale is a simple scale ranging from 1 to 10.
- A pedometer (Yamax SW200 digi-walker) will be worn at the waist for two consecutive days on three occasions (baseline, week 6 and week 12) to record the number of steps taken per day. enough to be counted as a full day, 10 hours of monitoring as the minimum amount of time needed to identify a valid day will be used.

e) Health-related QoL: This will be assessed using the Short-Form-12 QoL instrument which is widely used measure of general health and QoL status.

f) Psychological Function: Tampa Scale for Kinesiophobia (TKS) The TSK will be examined to provide a brief fear of movement scale .

Participant Timeline

The anticipated timelines for the project are as follows:

- October 2020 Baseline testing commences
- April 2020 Recruitment complete
- June 2020 All participants complete immediate post intervention testing (Week 6)
- August 2020 All participants complete 3 month follow-up (Week 12)

Sample Size

Sample size calculation considered the physical function measure of KOOS result (change from baseline). A clinically meaningful difference is considered to be 15%. The margin was derived from a minimal important difference KOOS-pain subscale (MCID80 KOOS-PS) score of 10 units reported for the KOOS based on the study by Lyman in KOA patients. It was estimated that individual randomization would require 22 patients in each group participants per arm for a trial with 80% power to detect a 15% difference between trial arms, with a 5% significance level (2-tailed) and allowing for 20% withdrawal by 3 months. To take account of possible dropouts, we wanted to include 70 participants.

## Recruitment

We will recruit participants with painful KOA from the community of the West Attica premises. A number of recruitment strategies will be used including (i) advertising through then municipality, community centers, local newspapers, Peristeri KEP Ygeias and University websites, University staff newsletters, and Facebook; (ii) placing brochures and flyers and study posters in medical and physiotherapy clinics; (iii) conducting presentations about knee OA in the local community.

## Assignment of interventions-allocation

This will be an assessor-blinded, 2-arm randomised controlled trial of a 6-week intervention involving 2 physiotherapy visits together with home practice. Measurements will be taken at baseline, immediately following the intervention (6 weeks) as well as at 6 weeks following the end of the program to examine maintenance of any intervention effects (12 weeks). Specific assessment of adherence to the treatment program will also be made at weeks 2 and 6. The protocol will conform to CONSORT guidelines for reporting non-pharmacological interventions (Figure 1).

## Randomisation- Allocation concealment

The randomisation schedule will be prepared by the supervisor of the study (GG) using a computer generated random numbers table. Recruited participants will be randomly allocated to either the intervention group or control group at a 1:1 ratio. Random numbers will be generated using a computer software program run by an external statistician. Each of the random numbers with the group assignment will be written on a piece of paper and packed in a sealed envelope. After baseline assessment, participants will be provided an envelope according to the randomization sequence by a volunteer undergraduate who will prepare consecutively numbered, sealed, opaque envelopes. The envelopes will be kept in a locked location accessible only by the unblinded researcher (MM). The unblinded researcher will then schedule the participants’ first appointment with their treating physiotherapist.

The investigator (AK) will be blinded to the participant randomization assignment and will not be involved in providing the interventions. Participants will be requested not to disclose details about their treatment to the outcome assessors. The primary investigator (physiotherapist and researcher) is by necessity unblinded. The statistician will be blind to group allocation until completion of the statistical analyses.

## Data collection and management

## Confidentiality

Participants will be assigned to a unique digital trial code to ensure that treatment outcome measurement and statistical analysis will be performed blind to treatment allocation. Patient information will be stored in a separate database. Data collected will be stored for 3 years in the personal computer of the primary researcher and hardcopies will be safely stored in a locked drawer in the office of the primary researcher in the University campus.

Statistical methods for primary and secondary outcomes

Demographic and clinical characteristics as well as baseline data will be presented to assess the baseline comparability of the intervention groups. These variables will also be examined for those participants who withdraw from the study. Descriptive statistics will be presented for each group as mean change (standard deviation, 95% confidence intervals) in the two primary outcomes and all secondary outcomes from baseline to 12 weeks. Means and standard deviations for continuous variables (e.g., steps/day) and computed proportions for categorical variables (e.g., proportions meeting the time-intensity guidelines). Analysis of covariance (ANCOVA) will be performed using IBM SPSS version 19 for all the primary and secondary outcome measures with baseline measures as covariates. Between-group mean differences and 95% confidence intervals will be estimated with a mixed effects linear regression model. Categorization according to milestones of physical activity levels as well as adherence levels could be made and further subgroup analysis will be performed. In the case of missing data in the relevant directions according on how to handle missing data in every outcome measure will be followed.

**Gant Chart**

| **Months** | 0-3 | 6 | 12 | 18 | 24 |
|---|---|---|---|---|---|
| **Literature Review**<br><br>**Study Design and Protocol** | | | | | |
| **Approval from Ethics Committee** | | | | | |
| **Data Collection** | | | | | |
| **Data Analysis** | | | | | |
| **Writing** | | | | | |

Expected outcomes

This exploratory trial will show how a combination of exercise, education and outdoor activity can impact on knee pain levels following a 6-week program, as well the feasibility of maintaining PA up to 3 months. The novel findings will enable evidence-based data as to the efficacy of this option for the management and behavioral change towards PA of patients with KOA
