# Supplementary material for: How effective is a blended web-based rehabilitation for improving pain, physical activity, and knee function of patients with knee osteoarthritis? Study protocol for a randomized control trial
Source: PLoS One. 2022 May 26;17(5):e0268652. doi: 10.1371/journal.pone.0268652 (PMC9135189; doi:10.1371/journal.pone.0268652)
Supplement: Attachment — Submitted filename: Review_Blended web-based rehabilitation for knee osteoarthritis patients..docx [file pone.0268652.s003.docx]

Thanks for the opportunity given to me to review the article tittled: “*How effective is a blended web-based rehabilitation for improving pain, physical*

*activity, and knee function of patients with knee osteoarthritis?”*

**General comments**

In general, it is an interesting and duly justified study proposal for the most part. However, some aspects of the methodology do not seem entirely clear to me, so I would like the authors to resolve my doubts. In this section, I also believe that some aspects are explained in duplicate, or in a fragmented manner, as in the case of the walking routes, which makes it difficult to understand.

**Introduction**

Overall, a good introduction, with an appropriate structure and justifying the need for the intervention. Small comments to be made

Line 20. Another important limitation of the exercise-focused programs is the low degree of adherence that subjects with KOA tend to show. For this reason, other measures (motivational talks, calls, etc.) are often necessary to improve compliance with these programs.

Line 26. There are examples of studies carried out on subjects with KOA that have used a website or an electronic application as a support for teaching physical exercise. It would be appropriate for this paragraph of the introduction to include some reference to these studies, as well as note the possible effects it may have on subjects with KOA por increasing PA. In this way, this paragraph could be more targeted to the condition being studied.

**Material and methods**

Line 52: What does it mean "exploratory" in this context? Is this concept necessary?

Line 81: If the level of physical activity performed by each subject prior to the start of the intervention has not been assessed, this should appear as a limitation, or at least be mentioned in the discussion.

Line 95: You mention that both groups receive the introductory session, but please also specify that the control phone calls are also received by both groups.

Line 104: Please, add the reference

Line 126: During the introduction, the need for a blended intervention of physical activity and behavioral programs has been mentioned. However, when it comes to explaining the intervention of the experimental group, this structure is not so clear. I suggest that the authors partially rewrite this paragraph in such a way that the blended structure and how each of the components is worked on is clearer, so that the use of the term "blended" can be justified.

Usual care paragraph: To my understand, the walking journey that perform subjects in usual care group is also mutually agreed during the initial session, as explained before. Am I rigth?

However, it is not clear if they receive any explanation on how to make the walking journey, with what parameters and what factors were used to establish these characteristics, as was detailed in the experimental group. If they simply receive the advice to walk, please specify.

Any justification of why control group performed journey route 5 times per week and the experimental only 3 times?

Lines 154-155: It is already explained. Please, remove this sentence

**Discussion**

Lines 172-176: If pain was already assessed by the specific domain of the KOOS scale, is there any compelling reason to assess it with the NPRS week Average knee pain as well? Please, explain if it the case

Lines 245-249: I also understand that one of the objectives is whether this type of intervention contributes to increasing physical activity levels in people with KOA
